# Optic nerve sheath diameter sonography during the acute stage of intracerebral hemorrhage: a potential role in monitoring neurocritical patients

**DOI:** 10.1186/s13089-020-00196-1

**Published:** 2020-11-25

**Authors:** M. Bender, S. Lakicevic, N. Pravdic, S. Schreiber, B. Malojcic

**Affiliations:** 1 Department of Neurology, University Hospital Mostar, Bijeli Brijeg bb, 88 000 Mostar, Bosnia and Herzegovina; 2 Department of Neurology, Asklepios Kliniken, Brandenburg, Germany; 3 Department of Neurology, University Hospital Center Zagreb, Zagreb, Croatia

**Keywords:** Optic nerve sheath diameter, Intracranial hemorrhage, Ultrasound, Outcome

## Abstract

**Background:**

Optic nerve sheath diameter (ONSD) sonography has been proposed as a reliable bedside tool for the detection of increased intracranial pressure (ICP). ONSD reacts almost simultaneously to oscillations in ICP. The aim of this study was to investigate the ONSD dynamics in the acute stage of intracerebral hemorrhage (ICH) and to compare ONSD dynamics to the clinical outcome.

**Methods:**

We enrolled 35 acute ICH patients and 20 healthy volunteers in this prospective study. At the admission, all patients underwent brain CT scan and ONSD sonography. We repeated the ONSD on the second and the third day in all patients while CT scan was repeated if a patient condition deteriorated. The changes in serial ONSD measurements were termed as stable or unstable ONSD trend. ONSD trend was considered as unstable if variations of average ONSD were above 5%. The outcome of the patient was assessed with the Modified Rankin Scale (mRS) and Glasgow Outcome Scale (GOS).

**Results:**

In healthy volunteers serial ONSD recordings for 3 days revealed a stable trend in 100%. However, in the study group, 23 patients had unstable and 12 had stable ONSD trend during the acute stage of ICH. The patients with unstable ONSD trend were more likely to have worse outcomes (*p* value 0.003).

**Conclusion:**

In patients with ICH, the acute-phase ONSD dynamics can help in predicting the clinical outcome.

## Introduction

ONSD has been proposed as a reliable, non-invasive bedside tool for the detection of raised ICP. Increased ICP, transmitted through the subarachnoid space, causes ONSD enlargement, which can easily be detected using ultrasound (US) [1, 2]. This was first demonstrated by Hansen and Helmke in an experimental study on cadaveric specimens with the greatest degree of distension occurring at 3 mm behind the optic globe, whereupon this location has become a standard measurement point [3]. Since then, ONSD has been well studied in experimental and clinical settings, in healthy populations as well as in patients with elevated ICP of different etiologies. Quite a few studies have compared ONSD sonography to invasive ICP measurements and most of them proven a good correlation. Furthermore, they demonstrated that the ONSD reflects an immediate change in ICP and changes almost concurrently with rapid ICP variations [2, 4]. However, the optimal ONSD cutoff point for detection of increased ICP has not been well established, the range of proposed values varies from 4.8 to 5.9 mm [5–9]. Available data regarding normal ONSD are also inconclusive, with a wide inter-individual range of ONSD in the general healthy population [10–13]. Even though ONSD sonography is not as accurate as invasive ICP monitoring, many studies have demonstrated that ONSD reflects an immediate change in ICP, hence, serial ONSD recordings might be useful in monitoring the patients with elevated ICP when invasive monitoring is not possible or is not recommended. Accepting limitations of the method, we investigated the dynamics or relative ONSD changes rather than the absolute ONSD changes in the acute phase of spontaneous, non-traumatic ICH and we evaluated its association with clinical course and outcome.

## Methods

### Study population

This prospective observational study was carried out in University Hospital Mostar, over a period of 15 months from January 2018 to March 2019. The study was approved by the Ethics Committee of the hospital in accordance with the guidelines of the Declaration of Helsinki [14]. All participants provided written informed consent, for the unconscious patients, the informed consent was obtained by a proxy. All patients presenting to our hospital, no more than 6 h after the symptom onset, with a diagnosis of supratentorial non-traumatic ICH were enrolled in the study. Exclusion and inclusion criteria are listed in Table 1. A control group (*n* = 20) consisted of healthy volunteers, without a known intracranial or ocular pathology. The patients and the control group were not paired.

**Table 1:** Inclusion and exclusion criteria

Inclusion criteria	Supratentorial non-traumatic ICH confirmed by brain CT
	Admission to the hospital within 6 h after symptom onset
	≥ 18 years of age
	Brain neuroimaging (CT) within 6 h after symptom onset
Exclusion criteria	Ocular trauma or a disease involving the optic nerve
	Inability to preform US measurement within 1 h from brain CT

*ICH* intracerebral hemorrhage, *CT* computed tomography, *US* ultrasound

### Measurements—study protocol

The study protocol included at least two brain CT scan. First CT scan was done at the admission and second during the follow-up phase during the hospitalization. The brain CT scan was evaluated by an investigator blinded to other results, including ONSD measurements and clinical status. At the level of the third ventricle, midline shift was measured [15] and the ICH volume was assessed using a widely recognized ABCD/2 method [16]. Clinical status was evaluated daily using the National Institute of Health Stroke Scale (NIHSS) and Glasgow Coma Scale (GCS) performed simultaneously to ONSD measurement [17]. Intracerebral Hemorrhage (ICH) Score was also performed at the admission and the outcome of the patient was assessed when the patients were discharged from the hospital, using the mRS and the GOS [18, 19].

### Ultrasound examination

**Fig. 1:**
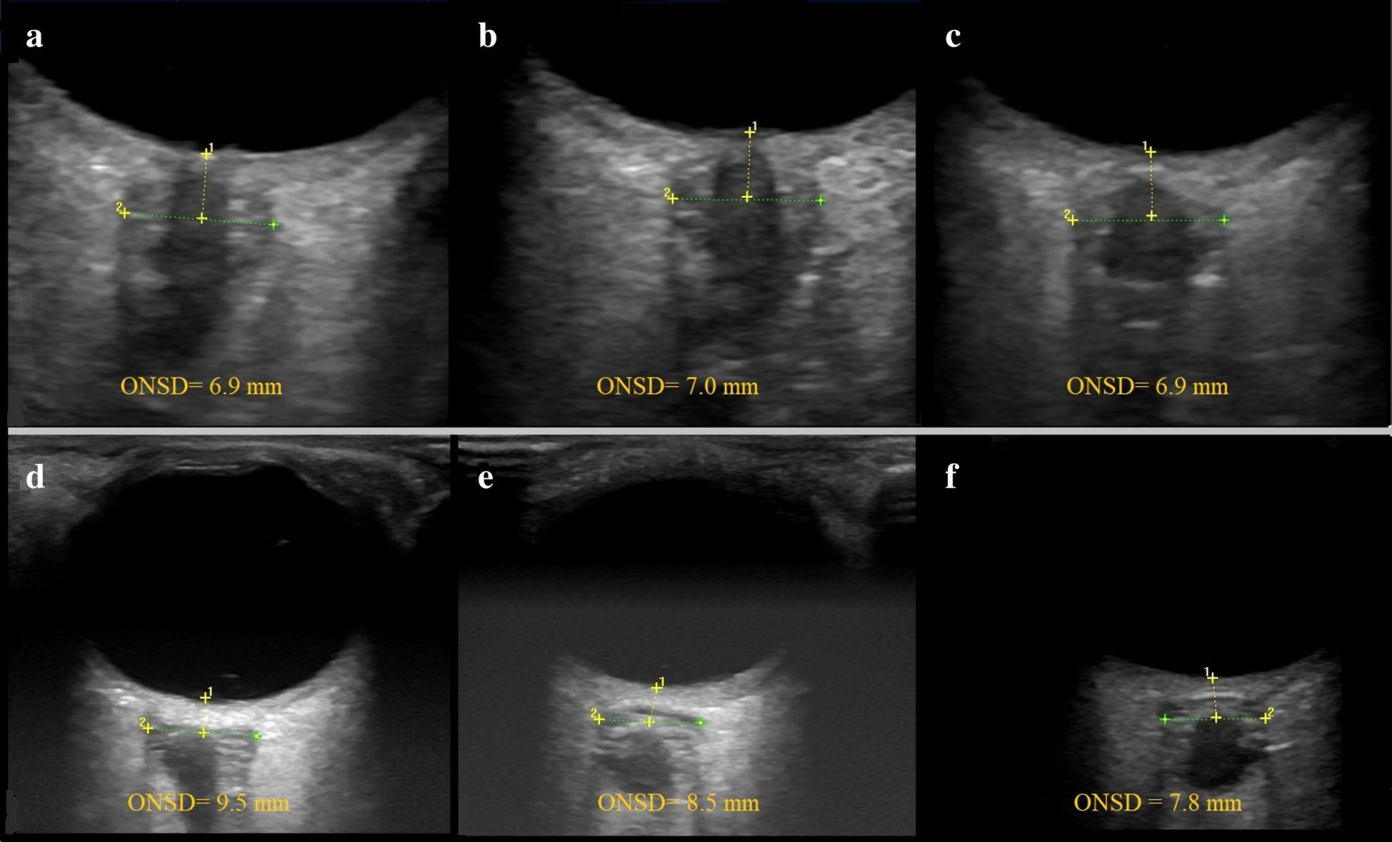
An example of different ONSD trends during the acute stage of ICH. ONSD ultrasound in a patient with ICH performed at admission (**a**), on the second (**b**), and the third day (**c**), representing the stable ONSD trend and example of unstable ONSD trend in another patient (**d**–**f**) (*ONSD* optic nerve sheath diameter)

Ocular sonography was performed at admission, on the second and the third day, by an investigator with previous experience in sonography measurements of the ONSD in more than 100 patients. Sonography was performed using a GE Logiq P6 scanner (General Electrics Medical Systems, Milwaukee, WI, USA) with a 13–10 MHz linear transducer. The patients were examined in Semi-Fowler’s position. They were asked to try to suppress eye movements. If the patient was unconscious and not able to fix on primary gaze, a slight adjustment of angulation was required. The insonation program was set to “small parts” or “superficial” with an insonation depth set to 5–8 cm and with optimally adjusted gain to obtain good contrast between the structures. The mechanical index was set to 0.2. ONSD was measured 3 mm behind the globe. Video of every ultrasound was recorded and analyzed later by a single-blinded investigator. Each measurement was performed three times and the mean value was calculated afterward. Changes in serial ONSD measurements were termed as stable or unstable ONSD trend. We considered that the patient has an unstable ONSD trend if the variations (increase or decrease) of average ONSD (left or right) were above 5% (Fig. 1) [20].

### Statistical analysis

Categorical variables were presented as frequencies and percentages, and numerical variables were presented as medians either with interquartile range or confidence intervals. The differences between categorical groups were tested using Chi-squared, while the differences between numerical values were tested using the Mann–Whitney *U* test.

## Results

During the study period, 35 patients met the inclusion criteria. Clinical and demographic data are shown in Table 2. The patient group and the control group did not significantly differ in terms of gender (*χ*^2^ = 0.012, *P* = 1.00). However, groups differed significantly in age (*χ*^2^ = 55.0, *P* < 0.001). Patients were significantly older. Median binocular ONSD values in patients with acute ICH were significantly higher from those in the control group (*χ*^2^ = 55.0, *P* = 0.041). The median intra-observer variation was ± 0.1 mm (ICC = 0.99, *P* < 0.001, 95% CI 0.99–1.00). In healthy volunteers, serial ONSD recordings revealed a stable trend in 100%. However, in the study group, 23 patients had unstable and 12 had stable ONSD trend during the acute stage of ICH. Serial ONSD sonography recordings during the acute stage of ICH are shown in Table 3. The patients in the unstable ONSD group trend were more likely to have higher ICH scores, ICH volume, mRS, and lower GCS and GOS. On the other hand, no correlation was demonstrated between ONSD trend and midline shift, localization of ICH, NIHSS, and ONSD at admission (Table 4). There were no differences between left and right ONSD in the healthy volunteers, but 11 patients had one ONSD significantly larger (> 5%) than the other and in 8 of 11 cases larger ONSD was on the same side as the ICH.

**Table 2:** Demographic and clinical data in study and control groups

	Acute ICH (*n* = 35)	Healthy volunteers (*n* = 20)	*P*-value*
Age [median (IQR)] (years)	74 (66–79)	30.5 (27–36)	< 0.001
Male sex [n (%)]	24 (69.0)	14 (70.0)	1.00
NIHSS score at admission [median (IQR)]	13 (9–18)		
GCS at admission [median (IQR)]	12 (11–15)		
ONSD [median (IQR)] (mm)	7.1 (6.6–7.5)	6.2 (6.0–6.7)	0.041
ICH volume (cm^3^) [median (IQR)]	2 (1–3)		

*GCS* Glasgow Coma Scale, *ICH* intracerebral hemorrhage, *NIHSS* National Institute of Health Stroke Scale, *ONSD* optic nerve sheath diameter, *IQR* interquartile range

*Mann–Whitney continuous data test Chi-square for categorical data

**Table 3:** ONSD serial recordings during the acute stage of ICH

Patient (n), age (years), sex	Time since admission (days)	ONSD (mm)			ONSD trend	ICH volume (cm^3^); midline shift (mm)	GCS	NIHSS	ICH score	MrS; GOS
		Right	Left	Average						
1 91, F	0	7.03	7.10	7.06	Unstable (ascending)	31; 8	6	30	4	6; 1
	1	7.10	7.30	7.20			6	30		
	2	7.70	7.76	7.73			6	30		
2 58, F	0	5.56	5.33	5.44	Unstable (ascending)	43; 6	6	28	3	6; 1
	1	5.83	6.10	5.96			6	28		
	2	6.06	5.80	5.93			6	28		
3 76, M	0	6.73	6.03	6.38	Stable	94; 9	15	13	1	6; 1
	1	6.46	5.96	6.21			15	12		
	2	6.73	5.93	6.33			15	12		
4 83, M	0	7.60	7.20	7.40	Stable	11; 4	15	12	1	4; 4
	1	7.23	7.26	7.22			15	12		
	2	7.30	7.23	7.25			15	10		
5 61, M	0	8.16	7.85	8.05	Stable	25; 2	15	6	0	2; 5
	1	8.20	7.75	7.98			15	6		
	2	7.93	7.80	7.86			15	5		
6 79, F	0	7.10	6.93	7.01	Stable	69; 7.3	8	31	3	6; 1
	1	6.96	6.90	6.93			9	31		
	2	7.00	6.86	6.93			9	31		
7 59, F	0	6.76	6.66	6.71	Stable	37; 6	15	13	2	4; 3
	1	6.66	6.56	6.61			15	11		
	2	6.63	6.63	6.63			15	11		
8 65, M	0	7.10	6.96	7.03	Stable	10; 6	15	7	1	4; 4
	1	7.07	6.80	6.94			15	6		
	2	7.00	6.70	6.85			15	6		
9 62, M	0	7.70	7.63	7.66	Stable	10; 0	15	11	0	4; 3
	1	7.90	7.60	7.75			15	10		
	2	7.63	7.50	7.56			15	10		
10 71, M	0	7.50	7.60	7.55	Unstable (oscillating)	71; 8	4	31	4	6; 1
	2 h*	7.40	6.55	6.80			3	31		
	1	7.90	6.50	7.20			3	31		
	2	6.55	6.55	6.55			3	31		
11 82, M	0	6.50	6.85	6.68	Unstable (descending)	49; 8	12	12	4	6; 1
	1	6.6	6.90	6.55			12	12		
	2	6.0	6.66	6.43			12	12		
	3	5.65	6.50	6.08			12	12		
12 77, F	0	8.56	8.10	8.33	Unstable (descending)	13; 7	11	11	2	4; 3
	1	7.86	8.10	7.98			11	11		
	2	8.20	7.66	7.93			12	11		
13 88, F	0	6.50	6.55	6.52	Unstable (descending)	13; 7	12	7	3	5; 3
	1	6.56	6.55	6.54			12	7		
	2	6.45	5.75	6.10			13	7		
14 72, M	0	6.96	7.40	7.18	Unstable (descending)	8; 0	15	4	1	1; 5
	1	7.25	7.16	7.20			15	4		
	2	6.10	6.75	6.45			15	3		
15 67, F	0	7.13	7.20	7.16	Unstable (ascending)	34; 9	12	15	3	5; 3
	1	7.26	7.80	7.53			12	15		
	2	7.23	7.56	7.41			12	15		
16 71, M	0	7.60	7.40	7.50	Unstable (ascending)	78; 5	11	11	3	6; 1
	1	7.73	8.13	7.93			11	12		
	2	7.46	7.80	7.63			11	12		
17 55, M	0	6.96	7.23	7.10	Unstable (descending)	76; 11	12	13	3	6; 1
	2 h*	6.06	6.50	6.28			3	30		
	1	6.36	6.36	6.36			3	30		
	2	6.26	6.70	6.46			3	30		
18 72, F	0	6.00	7.10	6.55	Unstable (ascending)	31; 0	15	3	1	2; 4
	1	6.93	7.60	6.98			15	3		
	2	7.40	7.65	7.52			15	3		
19 74, M	0	6.96	7.50	7.23	Unstable (descending)	66; 14	10	16	3	6; 1
	1	7.10	7.60	7.35			11	16		
	2	6.66	6.63	6.64			11	15		
	3	6.73	7.10	6.91						
20 67, M	0	7.20	7.46	7.33	Unstable (oscillating)	30; 5	12	17	3	6; 1
	1	7.70	8.20	7.95			12	17		
	2	7.20	7.73	7.26			12	17		
	3	7.60	7.60	7.60						
	4	6.96	7.65	7.30						
	5	6.96	7.50	7.22						
21 76, M	0	7.26	8.23	7.75	Unstable (descending)	31; 0	11	9	2	2; 4
	1	7.30	7.90	7.60			15	4		
	2	7.30	7.66	7.48			15	1		
22 74, M	0	6.30	6.43	6.36	Unstable (descending)	62; 7	12	19	3	6; 1
	1	6.26	6.60	6.43			12	19		
	2	7.23	6.86	7.05			12	19		
23 66, M	0	6.66	6.56	6.61	Unstable (descending)	33; 6	12	18	3	6; 1
	1	6.30	6.16	6.23			12	18		
	2	6.35	6.05	6.27			12	18		
24 63, M	0	6.36	6.20	6.28	Stable	1; 0	15	5	0	1; 5
	1	6.40	6.16	6.28			15	4		
	2	6.36	6.10	6.23			15	4		
25 80, F	0	7.10	6.90	7.00	Unstable (ascending)	50; 9	7	29	4	6; 1
	1	7.63	7.53	7.58			7	29		
	2	7.60	7.53	7.56			7	30		
26 61, M	0	6.63	6.96	6.79	Unstable (descending)	5.8; 0	15	10	0	4; 3
	1	6.06	6.45	6.25			15	10		
	2	5.6	6.53	6.08			15	8		
27 83, F	0	6.60	6.56	6.58	Stable	7; 7	15	17	1	4; 3
	1	6.66	6.46	6.56			15	17		
	2	6.80	6.70	6.80			15	17		
28 77, M	0	7.86	7.20	7.53	Unstable (oscillating)	8.3; 0	15	12	0	5; 3
	1	8.16	6.66	7.41			15	12		
	2	8.35	7.03	7.69			15	12		
29 67, M	0	9.16	9.70	9.43	Unstable (descending)	97; 13	5	31	4	6; 1
	1	8.86	9.66	9.26			4	31		
	2	8.2	9.33	8.79			4	31		
	3	8.30	8.66	8.48			4	31		
	4	8.00	8.00	8.00			4	31		
30 78, M	0	7.46	8.25	7.85	Stable	1; 0	15	5	0	2; 5
	1	7.36	8.30	7.83			15	5		
	2	7.46	8.36	7.91			15	5		
31 79, M	0	7.10	7.00	7.05	Stable	4; 0	15	6	0	1; 5
	1	7.00	7.00	7.00			15	6		
	2	7.10	7.10	7.10			15	6		
32 82, M	0	7.46	7.40	7.43	Unstable (oscillating)	30; 8	12	15	4	6; 1
	1	8.16	8.06	8.11			12	16		
	2	7.96	7.56	7.76			12	16		
33 76, F	0	7.13	7.33	7.23	Unstable (descending)	75; 14	4	31	4	6; 1
	1	7.16	6.53	6.84			4	31		
	2	7.05	6.66	6.85			4	31		
34 87, M	0	6.66	6.60	6.63	Stable	31; 6	15	11	2	2; 4
	1	6.66	6.60	6.63			15	11		
	2	6.90	6.90	6.90			15	11		
35 71, M	0	6.80	7.55	7.18	Unstable (oscillating)	40; 5	14	13	2	6; 1
	1	7.46	7.26	7.36			14	13		
	2	7.50	6.66	7.08			14	13		

*ICH* intracerebral hemorrhage, *ONSD* optic nerve sheath diameter, *M* male, *F* female

^*^Placement of intraventricular catheter

**Table 4:** Comparison between patients with stable and unstable ONSD trend

	Median (95% CI)		
	Stable ONSD trend	Unstable ONSD trend	*P*-value*
Number	12	23	
ONSD admission	7.0 (6.6–7.6)	7.2 (6.9–7.4)	0.532
mRS	4.0 (2.0–4.0)	6.0 (5.0–6.0)	0.003
GOS	4.0 (3.0–5.0)	1.0 (1.0–3.0)	0.002
ICH score	1.0 (0.0–1.9)	3.0 (2.4–3.7)	< 0.001
GCS	15.0 (15.0–15.0)	12.0 (10.4–12.0)	< 0.001
NIHSS	11.0 (6.0–13.0)	15.0 (11.4–18.7)	0.073
ICH volume (cm^3^)	10.5 (4.5–35.9)	34.0 (30.3–57.8)	0.029
Midline shift (mm)	5.0 (0.0–6.8)	7.0 (5.0–8.0)	0.079

*ONSD* optic nerve sheath diameter, *mRS* Modified Rankin Scale, *GCS* Glasgow Coma Scale, *ICH* intracerebral hemorrhage, *NIHSS* National Institute of Health Stroke Scale, *GOS* Glasgow Outcome Scale

*Mann–Whitney continuous data test Chi-square for categorical data

## Discussion

To the best of our knowledge, this work for the first time provides information about the dynamics of ONSD measured by means of ultrasound in the acute stage of ICH. We demonstrated that in some ICH patients ONSD values change significantly during the acute stage, whether they rise or fall, and those patients are more likely to have worse functional outcomes when compared to ICH patients with stable acute-phase ONSD values.

ONSD sonography seems to be a reliable surrogate of ICP, with high intra- and interobserver reliability [11, 21, 22] and good correlation between US and MRI measurements of the ONSD [23]. However, most of the published studies rely on a single ONSD measurement or the absolute values of ONSD for ICP diagnosis, rather than on serial recordings and relative changes that might be more useful.

Previous studies have demonstrated that ONSD changes concurrently with rapid ICP variations. Consequently, we could detect changes in ICP by detecting changes in ONSD values. This makes serial ONSD sonography a good non-invasive alternative for ICP assessment in neurocritical patients in whom invasive monitoring is not available or not indicated.

Only a few studies have investigated the utility of serial ONSD measurements. Toscano et al. performed daily ONSD measurements in 21 sedated neurocritical patients who eventually developed brain death (BD) and observed that ONSD values were increasing over time with the widest values occurring just after the BD. The increase of the ONSD was constantly correlated with elevated values of ICP [24]. Thotakura confirmed the utility of serial ONSD measurements in 40 patients with TBI who were in a conservative management program at the time of admission. All the patients with the descending trend had a good outcome and required no surgery, on the other hand, out of four patients who died, three were in ascending trend and the other in static trend [20]. Daily ONSD monitoring has proven to be useful in monitoring pediatric patients during diabetic ketoacidosis management as well as in monitoring patients with MCA infarction who are at risk of developing malignant MCA syndrome [25, 26]. Skoloudik et al. investigated the utility of ONSD sonography during the acute stage of ICH. They emphasize that relative enlargement of the ONSD is a more sensitive indicator of raised ICP than the absolute ONSD value [27]. Naldi et al. also investigated ONSD in patients with ICH and have shown that the second assessment of ONSD measurements may be useful in identifying patients with the persistency of elevated ICP [28]. Both studies have indicated the need for more research in this field.

Furthermore, we demonstrated that ONSD reacts almost simultaneously to oscillations in ICP. Two patients underwent placement of external ventricular drain (EDV) and ONSD descending trend appeared right after. The average left and right ONSD at admission for patient 10 and patient 17 (Table 3) were 7.55 mm and 7.10 mm, respectively. Only a few minutes after the placement of EDV, sonography of ONSD was done again, this time showing a significant decline in ONSD values, on 6.80 mm for patient 10 and 6.28 mm for patient 17. This could be one more proof that ONSD reacts to ICP changes in real-time, especially because ONSD significantly decreased immediately after the placement of EDV.

The values of ONSD in a healthy subject in this study were higher than has usually been found previously (median 6.2 mm; 95% CI 6.1–6.7). This could be due to multiple reasons including ethnic diversity and genetic difference as well as measuring the technique of ultrasound and expertise of the observer.

This study has some limitations. The lack of ICP data makes it impossible to correlate ONSD values with the actual ICP. Although the sample size in the study was sufficient for adequate statistical analysis, the number of participants was relatively small and future studies with a larger number of patients are needed to strengthen our results. One of the limitations was that there was no uniform time of control neuroimaging. Control brain CT was not necessarily done during the first 2 days which is the period in which we monitored ONSD. Consequently, a comparison of the ONSD dynamics with the dynamics of intracranial status would not be valid. Control CT scan was available for 32 patients (3 died before follow-up brain CT). 10 patients had significant worsening of intracranial status in terms of increased ICH volume, perifocal edema, or progression of midline shift, and 9 of these 10 patients had an unstable ONSD trend (3 oscillating, 3 ascending and 3 descending trends).

Furthermore, the ONSD trend was marked as stable or unstable, but in the unstable ONSD group, there were patients with clear ascending, descending, and oscillating trends (alternating increase or decrease value) (Table 3). The sample size was not sufficient enough for adequate statistical analysis among these subgroups of the unstable trend. Mean mRS for oscillating, ascending, and a descending trend was 5.8, 5.2, and 4.8, respectively. Mean GOS values for these subgroups of ONSD unstable trend also differ similarly: 1.4 for oscillating, 1.8 for ascending, and 2.1 for descending trend. The patients with oscillating ONSD trends were most likely to have a worse outcomes, followed by ascending and then a descending trend.

Although the acute phase ONSD dynamics can help in predicting the clinical outcome in ICH patients, this study does not address whether ONSD trend improves prognostication over already known variables.

## Conclusion

The role of daily ONSD sonography in the acute stage of ICH has not yet been investigated. According to our results, the assessment of acute-phase ONSD dynamics can help predicting the outcome in ICH patients and serial ONSD assessment may be a useful noninvasive tool for the detection of ICP changes in neurocritical patients without invasive monitoring.

## Data Availability

The datasets used and analyzed during the current study are available from the corresponding author on reasonable request.

## Abbreviations

EDV: External ventricular drain
NIHSS: National Institute of Health Stroke Scale
GCS: Glasgow Coma Scale
ONSD: Optic nerve sheath diameter
ICP: Intracranial pressure
ICH: Intracerebral hemorrhage
mRS: Modified Rankin Scale
GOS: Glasgow Outcome Scale
CT: Computer tomography
US: Ultrasound
